# Revealing the Impact of Micro-SiO_2_ Filer Content on the Anti-Corrosion Performance of Water-Borne Epoxy Resin

**DOI:** 10.3390/polym15153273

**Published:** 2023-08-02

**Authors:** Bifeng Fan, Junjie Yang, Lin Cao, Xiao Wang, Jie Li, Yingfei Yang, Qiwei Wang, Peng Zhang, Florin Vogel, Wei Li, Zhidan Lin

**Affiliations:** Institute of Advanced Wear & Corrosion Resistant and Functional Materials, Jinan University, Guangzhou 510632, China; fanbf1997@163.com (B.F.); junjieyang0612@gmail.com (J.Y.); caol93@jnu.edu.cn (L.C.); wangxiao828@stu2021.jnu.edu.cn (X.W.); lijiehigh@163.com (J.L.); yfyang@jnu.edu.cn (Y.Y.); wangqiwei@jnu.edu.cn (Q.W.); lwxasn@sohu.com (W.L.)

**Keywords:** water-borne epoxy resin, micron SiO_2_ filler, corrosion resistance, AC-DC-AC test

## Abstract

Due to green development in recent years, water-borne epoxy resins (WBE) have become increasingly popular since they generate the lowest level of volatile organic compounds (VOC) during curing. However, because of the large surface tension of water, it is easy to produce voids and cracks during the curing process of the coating. An electrochemical strategy was used in this study to assess the impact of different SiO_2_ content on the corrosion performance of a WBE coating, in which micron spherical SiO_2_ particles were synthesized in a liquid phase reduction. The results showed that the synthesized micron spherical SiO_2_ particles were about 800 ± 50 nm in diameter and in an amorphous state. By hydrophilizing the surfaces of these SiO_2_ particles, uniform dispersion in an aqueous solvent and a WBE can be achieved. It is important to note that adding a small or excessive amount of SiO_2_ to a coating will not improve corrosion resistance and may even reduce corrosion resistance. With the appropriate modification of SiO_2_, corrosion resistance of composite coatings is greatly enhanced, as is the adhesion between the coatings and the metallic substrates. Because the appropriately modified SiO_2_ can effectively fill the pores that are formed during the curing process, a corrosive medium is less likely to react with the matrix when the medium comes into contact with the matrix. Based on their incorporation content of 3 wt.%, their corrosion resistance is the best after 16 cycles of AC-DC-AC accelerated corrosion tests.

## 1. Introduction

Over 2.2 trillion dollars are lost annually as a result of metal corrosion, approximately 3% of gross domestic product. Approximately half of this cost is attributed to corrosion prevention and control globally [1,2,3,4,5,6]. According to a study conducted in the US, metallic corrosion has a direct economic impact of approximately USD 276 billion per year, and corrosion control practices will reduce this cost by 15–35% [7,8]. Epoxy networks have been widely used in coatings among different thermosetting networks, and approximately 90% of metallic materials are protected by organic coatings to provide physical barriers against corrosion [9,10]. At present, polyurethane, phenolic resin, acrylic acid, and epoxy resin are commonly used anticorrosive coatings [11,12,13,14,15]. It is important to note that anticorrosive pigments are composed of various volatile organic compounds (VOCs) which may ingress into the environment during the production, application, or curing of the solvent-based coating formulations [16]. In contrast to other anti-corrosion coatings, water-borne epoxy resin (WBE) is a stable dispersion system in which an epoxy resin is dispersed as small particles or droplets in a continuous phase of water to reduce the use of VOCs as solvents and thinners. Due to the reduction of VOCs, it produces the least number of VOCs during curing [16,17,18]. In recent years, WBE has become increasingly popular due to its environmentally friendly characteristics [19,20,21]. In the film formation process, water’s high surface tension will unavoidably form pores and microdefects at the surface of the coating during water evaporation. In addition, hydrophilic groups will cause the film to form channels that penetrate it. This channel enables water, dissolved oxygen, and electrolytes to pass through the coating directly into the metallic substrate. Corrosion will occur as a result of these media reacting directly with metallic substrates. During prolonged exposure to corrosive solutions, the epoxy matrix undergoes hydrolytic degradation, which also exposes the epoxy matrix to corrosive media. Therefore, it is more vulnerable to damage from the external environment during service [22]. Electrochemical corrosion is a common phenomenon, causing metallic structures to degrade as a consequence of electrochemical corrosion [23]. Physicochemical interactions between metals and their environments result in electrochemical reactions of oxidation and reduction. The transfer of charge must have taken place in order for electrochemical reactions to proceed. It is necessary for Fe^2+^ ions to migrate from the substrate into the polymer coating, as well as oxygen and water to migrate from corrosive media into the polymer coating [24]. As a result, electrostatic interactions occur between corrosion media and metal substrate interfaces, disrupting the coating-substrate interface. As a matter of particular significance, it is important to be aware that oxygen continuously reacts with the metal on the metallic substrate to produce corrosion products [25,26]. As a result, corrosion products produce a push-up effect on the coating, which results in a loss of adhesion between the coating and the metallic substrate. This causes bubbles to form when the coating is applied. Last but not least, this will reduce the protective effect of the coating on the metallic substrate. During the curing process, it is essential to investigate ways to reduce defects formed and fill up the pores in order to enhance the coating’s physical barrier and reduce ion emission. At present, there are two main modification methods for waterborne epoxy resin: copolymerization modification and blending modification [27]. Through copolymerization, new functional groups are added to the epoxy system to increase the degree of cross-linking and improve the compactness of the coating. Blending modification is the physical mixing of other fillers and epoxy resin, using different fillers to plug the pores produced in the curing process of the coating and playing the role of physical barrier so as to improve the compactness of the coating.

Introduction of inorganic nanoparticles within polymeric matrixes has been recognized as one of the most effective ways to minimize micro-defects and pores in coatings, resulting in enhanced corrosion resistance [28,29,30]. By using inorganic fillers, more polymeric matrix molecules could be linked, increasing the crosslinking density of epoxy resin coatings. Nanoparticles derived from inorganic materials have been used in polymer base coatings to improve their anticorrosion properties, including carbon nanotubes [31,32], graphene oxide [33], ZnO [34], and SiO_2_ [35]. In particular, environmentally friendly and low cost SiO_2_ particles, due to the silicon oxygen bond (Si-O-Si), are highly resistant to acids, alkalis, chemicals inertia, and oxidation resistance and have recently been receiving considerable attention in the field of corrosion protection [36,37,38,39,40]. An investigation of composite coatings with nano-SiO_2_ designed to protect carbon steel was undertaken by Li [41]. Among inorganic coatings, SiO_2_ can be modified by its surface-active agent to become more scattered. Oil epoxy resin E-51 was used as a basic material for the coating, and the surface of SiO_2_ can be modified by its surface-active agent. The results showed that coatings that contain 2 wt.% nano-SiO_2_ possess the greatest corrosion resistance, which can be measured with a steady impedance value of 10^8^ Ω·cm^2^ after immersion in NaCl solution of 3.5 wt.%. Palraj investigated wear and corrosion resistance behavior of nano-SiO_2_ composite epoxy coatings prepared by using the sol-gel method [42]. In this study, nano-SiO_2_ particles sized between 20 and 70 nm were used as fillers in the epoxy polyamide composite coating. Upon immersion in a solution containing 3 wt.% NaCl solution, the impedance value of the composite coatings was measured to be about 2.3 × 10^6^ Ω·cm^2^ after immersion. Through the use of micro-SiO_2_ and polydimethylsiloxane (PDMS), Ke prepared hydrophobic surfaces that exhibited a 155° water contact angle by using a facile drop-coating method [43]. In this experiment, octadecyl trichlorosilane was used to modify SiO_2_ to improve its dispersion in organic solution. Despite the fact that nano-SiO_2_ is a costly material, Anitha et al. developed a hydrophobic coating without fluorine that is stabilized at the air-water interface and that was exhibited to possess exceptional corrosion resistance when exposed to salt spray for more than 1000 h [44]. These studies were mainly conducted with the modification of nano-SiO_2_ and micro-SiO_2_ for better dispersal with oil epoxy resin, but as more attention has been paid to environmental protection, water-based epoxy resin now tends to be used instead of oil-based epoxy. In spite of this, there are limited studies examining the effects of adding different concentrations of SiO_2_ to water-based epoxy resin in order to increase their corrosion resistance.

In this study, different amounts of micro-SiO_2_ were used to determine how much micro-SiO_2_ was required to maximize anticorrosion properties of WBE coatings. SiO_2_ particles with a dimension of 800 ± 50 nm were synthesized and then subjected to hydrophilic modification to achieve uniform dispersion in the epoxy coating independently. A scanning electron microscope (SEM) and Fourier transform infrared spectroscopy (FT-IR) analysis were used to characterize the morphology and the changes of function groups on the SiO_2_ surface before and after modification. Instead of using traditional EIS tests to determine the corrosion resistance of composite coatings, AC-DC-AC tests have been recognized as a method of determining corrosion resistance of composite coatings. In order to determine the best anticorrosive properties of modified SiO_2_, both macroscopic and microscopic morphologies of the cross-section of the samples were evaluated after AC-DC-AC testing. As a final step, we conducted scratch tests on composite coatings with varying SiO_2_ content on metallic substrates before and after the electrochemical test.

## 2. Materials and Methods

### 2.1. Materials and Coating

Tetraethyl orthosilicate (TEOS, (C_2_H_5_O)_4_Si, >99%), Cetyltreimthylammonium (CTAB, C_19_H_42_BrN, >99%), Dodecylamine (DDA, CH_3_(CH_2_)_11_NH_2_, >99%), and isopropanol (IPA, C_3_H_8_O, >99%) were purchased from Aladdin Industrial Corporation. γ-aminopropyl triethoxysilane (KH550, C_23_H_24_N_6_O_5_S_2_, >99%) was provided by Yuanye Biochemistry Technology Co. (Shanghai, China). The curing agent (BH-532) and epoxy resin (BH-653) were purchased from Dark Horse Chemical Co. (Dongguan, China). Information about the curing agent and epoxy is provided in Table 1 and Table 2. Q235 carbon steel (50 mm × 50 mm × 1 mm) is used as the base material, and it was polished with 400# abrasive paper and then cleaned in ethanol and via ultrasonic vibration.

### 2.2. Synthesis of the Modified Spherical SiO_2_

The solution-gel method is used to synthesize spherical SiO_2_. TEOS is used as a silicon source, and the CTAB and DDA are used as a surfactant. A homogeneous solution was prepared by dissolving 0.3 g of CTAB and 3.0 g of DDA in a mixture with 200 mL of ethanol, 80 mL of deionized water, and 60 mL of IPA until homogeneously dissolved. Then, the pH was adjusted with a 25% ammonia solution. A continuous stirring process was performed in the reactor in preparation for the next step. In addition, 10 mL of TEOS was added dropwise to the above compound, which was stirred continuously for 4 h at room temperature. Following centrifugation at 6000 rpm/min, the mixture was washed four times with ethanol and then dried under a vacuum for twelve hours at 70 °C.

The KH550 is used as a surface modifier to increase the dispersion of SiO_2_ in WBE. Self-synthesized SiO_2_ was added to the mixed solution of 100 mL ethanol and 4.3 mL KH550. A centrifuge was used to disperse the compound at a speed of 6000 rpm/min after stirring for four hours. The precipitate was poured into an appropriate amount of ethanol by using ultrasonication for 20 min and then repeated three times to remove the excess KH550. The sediment was dried in an oven.

### 2.3. Preparation of Composite Coating with Different Content of SiO_2_

**Figure 1:**
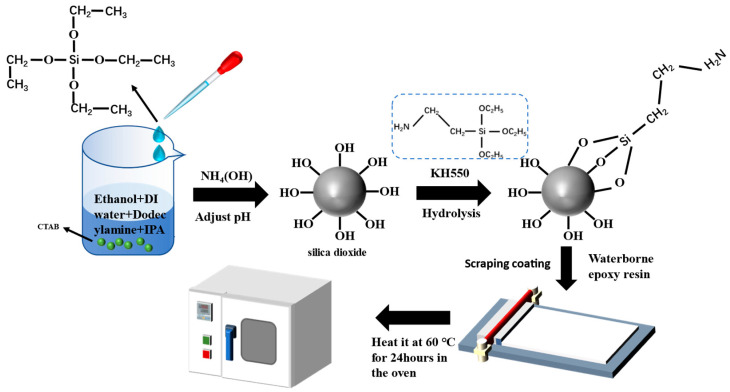
Schematic of synthesis of SiO_2_ and preparation of WBE resin coating.

Next, 1 wt.%, 3 wt.%, 5 wt.%, and 10 wt.% modified SiO_2_ was completely dispersed in 3 mL of deionized water with ultrasonic vibration, followed by 3 g of aqueous epoxy resin being thoroughly mixed with the SiO_2_ dispersion. To the above epoxy resin component, 3 g of the curing agent was added and mixed thoroughly. To ensure uniform coating thickness, the compound was applied directly to pretreated Q235 carbon steel and allowed to cure for 48 h at room temperature. In the course of curing, the mixture gradually changed from white to colorless. Furthermore, a neat epoxy coating was fabricated as a comparison. Upon curing, the coating had a thickness of approximately 120 μm ± 12 μm. This coating was prepared under the same conditions as the control group but without adding SiO_2_. According to the amount of modified SiO_2_ in the epoxy resin, different samples were referred to as EP-0, EP-1, EP-3, EP-5, and EP-10. Figure 1 illustrates the schematic diagram of the specific process.

### 2.4. Characterization of Modified SiO_2_

Field emission scanning electron microscope (FESEM, Zeiss, ULTRATM 55, Jena, Oberkochen, Germany) images of SiO_2_ particles and microstructures were taken. X-ray diffraction (XRD, Rigaku Ultima IV instrument, Tokyo, Japan) patterns were obtained by using monochromatic CuK_α_ radiation at a speed of 5°/min in the range of 5–80° at 40 kV and 20 mA. Fourier transform infrared spectroscopy (FT-IR, Nicolet iS50 + iN10, PerkinElmer, UK) was used to confirm the successful modification of SiO_2_.

### 2.5. Electrochemical Test

Gamry Framework electrochemical workstations equipped with three electrodes were used to collect the electrochemical data of a variety of samples immersed in NaCl solution of 3.5 percent. A saturated calomel electrode (SCE) was used as the reference electrode, a platinum plate with a surface area of 10 cm^2^ was used as the counter electrode, and the different samples were used as the working electrodes. During the measurement, a Faraday cage was placed inside the electrochemical cell to minimize external disturbances to the system.

Impedance spectra were recorded after an open circuit potential (OCP) test by using the Gamry Framework. During the AC-DC-AC test, all EIS tests were performed at frequencies ranging from 10^−1^ Hz to 10^5^ Hz.

**Figure 2:**
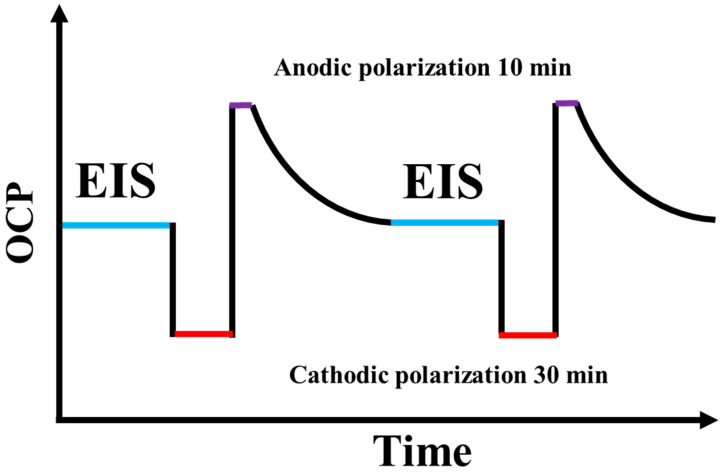
Schematic of AC-DC-AC test.

As a test method for observing the effect of adding SiO_2_ on the anticorrosion performance of the coating more quickly, the composite coating of SiO_2_ and epoxy resin was immersed in the NaCl solution of 3.5 percent. As soon as the OCP stabilized in 2 h, the AC-DC-AC test was performed as follows. In the first instance, the EIS was measured under OCP at this time, followed by cathodic polarization for 30 min at a DC voltage of −4 V and anodic polarization for 10 min at a DC voltage of +4 V, and then the system was relaxed again at the OCP until it returned to a steady state. Last but not least, we measured the EIS under an OCP that returned to steady state after a short period of time. A total of 15 cycles of AC-DC-AC tests were performed. The schematic diagram of an AC-DC-AC test is shown in Figure 2. For each sample, three parallel samples were prepared with different SiO_2_ additions. Using Zview software, the EIS data were fitted to electrical equivalent circuit models.

### 2.6. Surface Characterization

By using digital optical microscopy, the surface topography of the coated samples was recorded before and after the cross-orthogonal cycle. Scanning electron microscopes were used to examine the microscopic morphology of the coating in its cross-section under an electron accelerating potential of 1.5 kV.

The adhesion of composite coatings with different SiO_2_ content before and after the AC-DC-AC test was studied by using an automatic scratch testing machine (WS-2005, Lanzhou Zhongke Kaihua Science and Technology Development Co., Ltd., Lanzhou, China). The diamond indenter tip radius was 200 μm, and the cone angle was 120°. A linear load increase of 50 N per minute was set from 0 to 100 N. A scratch length of 4 mm was set.

## 3. Result and Discussion

### 3.1. Characterization of SiO_2_ and Composite Coating

**Figure 3:**
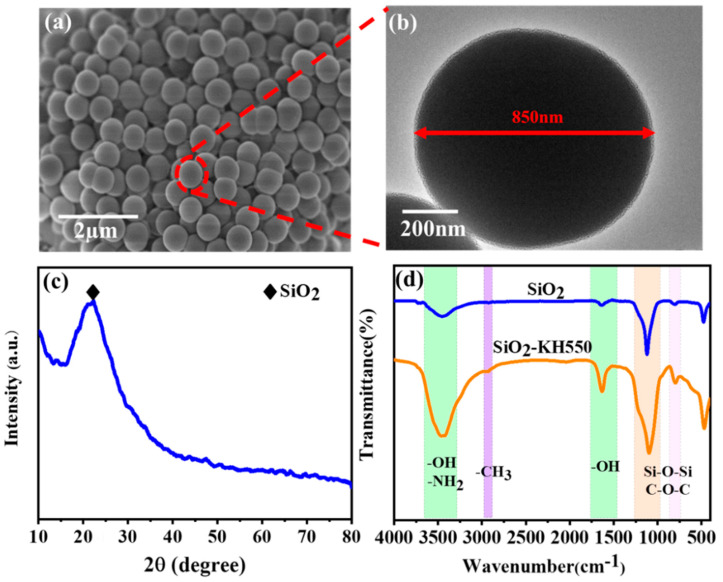
Morphology of the synthetic SiO_2_ (**a**) SEM images; (**b**) TEM images; (**c**) X-ray diffraction patterns of synthetic SiO_2_; (**d**) FT-IR spectra of synthetic unmodified SiO_2_ and the modified SiO_2_.

The morphology of the synthesized SiO_2_ particles was observed by using SEM. As shown in Figure 3a, the cluster of SiO_2_ particles reveals a regular spherical shape and quite a narrow distribution in particle size. To obtain the details of the SiO_2_ particles, ultrasonic dispersion was employed to get individual SiO_2_ particles for TEM observation. As shown in Figure 3b, the exemplified SiO_2_ particle displays an opaque morphology, which suggests that it could hardly be penetrated by the electron beam under the specific energy. In addition, the average diameter of the SiO_2_ particles was determined to be 800 ± 50 nm. An XRD analysis was performed on the synthesized SiO_2_ particles in order to determine their phase composition. As shown in Figure 3c, a typical broad peak is found at the vicinity of 21°, which suggests the amorphous state of the SiO_2_ particles synthesized via the modified Stober method [45,46,47,48].

Figure 3d exhibits the FT-IR spectra of SiO_2_ particles before and after surface modification. As can be seen, the absorption peak at 1104 cm^−1^ and 802 cm^−1^ represents vibrations of Si-O-Si bonds, and the absorption peak at 3449 cm^−1^ and 1629 cm^−1^ recirculates with vibrations of -OH bonds [49,50,51]. As a result of the hydrogen bond between water molecules and SiO_2_ for the peaks observed at around 1646 cm^−1^, it may be possible to demonstrate that the process of synthesis of the SiO_2_ is responsible for the observed peaks. SiO_2_ modified by KH550 displayed characteristic absorption peaks at 3449 cm^−1^ (-OH), 1629 cm^−1^ (-OH), and 1074 cm^−1^ (C-O-C) compared with self-synthesized SiO_2_. It is likely that the enhancement of this peak at 3449 cm^−1^ is a consequence of the -NH_2_ in KH550, as the introduction of amino groups can form hydrogen bonds with water molecules, thus increasing the hydrophilicity of SiO_2_ and promoting their good dispersion in aqueous epoxy resin. A new absorption peak was observed at 2924 cm^−1^ as a result of the stretching vibration peaks on the -CH_3_ in KH550 [52,53,54]. KH550 was grafted successfully onto SiO_2_ based on such results. Figure S1 shows the dispersibility of SiO_2_ in water before and after modification and various concentrations. According to Figure S1, the different contents of SiO_2_ immersed in water after 24 h have different dispersibility and stability. The unmodified SiO_2_ shows an obvious sediment in the water. In contrast, KH550 modified SiO_2_ (1 wt.% and 3 wt.%) can be uniformly suspended in water and stood for 24 h. This is primarily due to the formation of hydrogen bonds that were formed between the -NH_2_ on the surface of modified SiO_2_ and the -OH in water. Then, the water with a content 5 wt.% and 10 wt.% SiO_2_ also displays different degrees of settlement. Therefore, as the SiO_2_ content increases, the dispersibility in aqueous solution will decrease. Due to its internal structure of siloxane, and its surface consisting of dihydroxyl groups, isolated hydroxyl groups, and adjacent hydroxyl groups, SiO_2_ itself is easy to reunite due to its large number of hydroxyl groups [55]. In contrast, hydrogen bonds are formed when amino groups grafted onto SiO_2_ modified by KH550 are bonded with water molecules, thereby improving the dispersibility of SiO_2_. However, when too much SiO_2_ is added, the strong electrostatic attraction of the hydroxyl groups on silicon’s surface will cause the SiO_2_ to agglomerate into large particles which will deposit as a result of their own weight.

**Figure 4:**
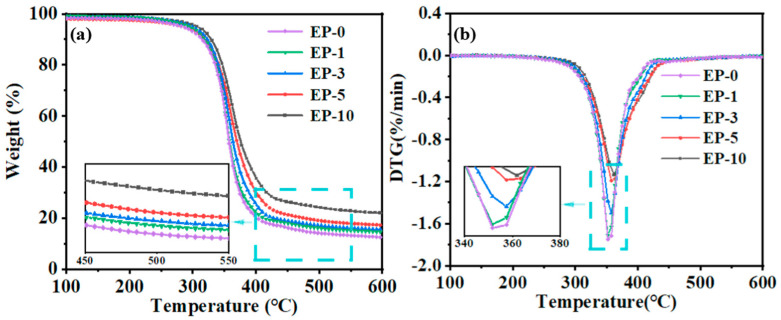
(**a**) Thermogravimetric (TGA) curves and (**b**) thermal decomposition rate (DTG) curves of the coating with different content of the modified SiO_2_.

Figure 4 illustrates both the thermal analysis curve for a coating without fillers and the composite coatings containing various concentrations of SiO_2_. Based on Figure 4a and Table 3, it can be seen that the final residual content of the composite coating increased with the theoretical content increasing, and because the melting point of SiO_2_ is 1700 °C, its weight will not change at 600 °C. This proves that the theoretical addition value of SiO_2_ is close to the actual addition. It should also be noted that the maximum decomposition temperature (*T*^d^_peak_) is an important indicator to evaluate the thermal stability of a composite coating [56,57]. With the addition of SiO_2_, the maximum decomposition temperature increases, and it may be evident that the thermal stability of the coating gradually improves when the SiO_2_ content increases as shown in Figure 4b [58]. When the waterborne epoxy resin is modified with -NH_2_ on the surface of SiO_2_ and dehydration condensation of -COOH in the waterborne epoxy resin, the compactness and glass transition temperature of the composite coating can be improved as a result of the Si-O-Si bonds in SiO_2_ and the high degree of cross-linking between KH550 and epoxy resin. Therefore, the addition of the modified SiO_2_ can enhance the coating’s stability.

**Figure 5:**
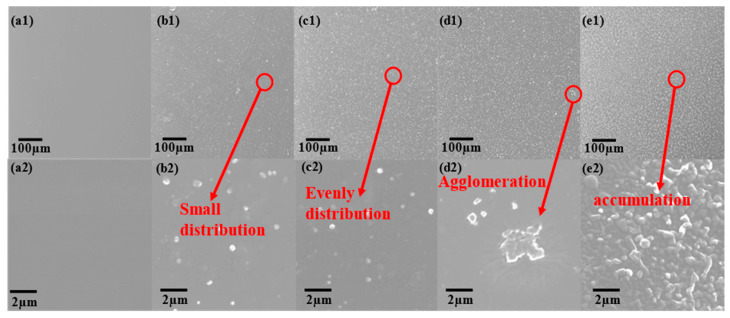
Surface morphology of the composite coating (**a1**,**a2**) EP-0; (**b1**,**b2**) EP-1; (**c1**,**c2**) EP-3; (**d1**,**d2**) EP-5; (**e1**,**e2**) EP-10.

A comparison of epoxy resin and composite coating with different levels of modified SiO_2_ is shown in Figure 5. According to Figure 5(a1), the surface of the pure epoxy coating is relatively smooth, whereas Figure 5(b1–e1) shows that the amount of modified SiO_2_ increases with increasing SiO_2_ content. It is possible to observe that SiO_2_ is evenly distributed over the surface of all composite coating. It can be shown in Figure 5(b1,c1) that SiO_2_ is evenly dispersed in the coating and also presents different dispersion states on the coating surface. However, there is an obvious agglomeration of fillers on the surface of the coating when SiO_2_ is added at an amount of 5 wt.% as shown in Figure 5(d2). However, when the addition of SiO_2_ was 10 wt.%, a large amount of SiO_2_ piled up on the coating surface.

### 3.2. Electrochemical Test

**Figure 6:**
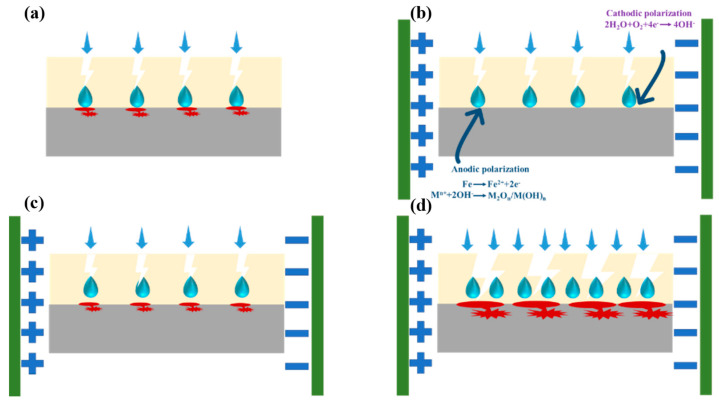
An illustration showing how corrosion damages the coating during AC-DC-AC test. (**a**) natural immersion; (**b**–**d**) In AC-DC-AC test.

The AC-DC-AC test is used in this study to assess the coating’s protective properties more rapidly. The difference between AC-DC-AC testing methods and natural immersion in composite coating is shown in Figure 6. Through the defects in the coating, the corrosion medium is brought into contact with the metallic substrate directly, and the reactions take place through cathodic and anodic reactions, resulting in corrosion as shown in Figure 6a. When the water penetrates the metallic substrate, oxygen reduction and alkalization occur through cathodic polarization, which pushes the coating away from the metallic substrate to promote the damage of the coating under the action of external electric field during AC-DC-AC tests. It has been observed that large quantities of oxides and hydroxides form on the metallic substrate during anodic polarization, further promoting the delamination between the coating and the metallic substrate.

**Figure 7:**
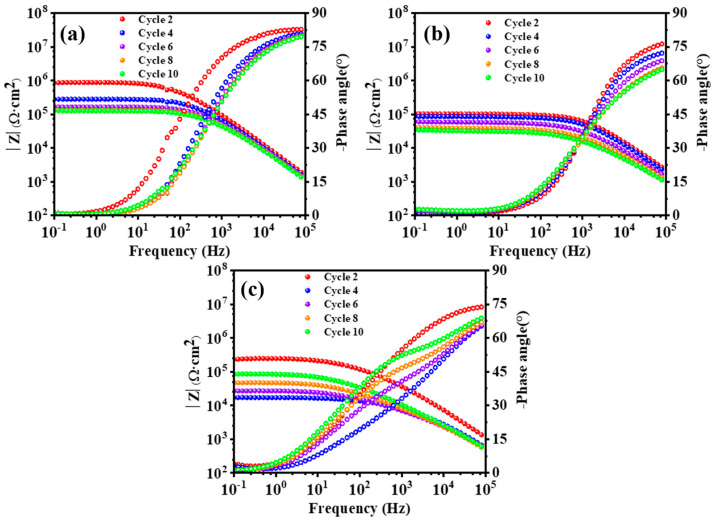
Bode plots of pure EP coating with different DC voltages used in AC-DC-AC test. (**a**) ±2 V; (**b**) ±3 V; (**c**) ±4 V.

In order to regulate the degradation of the WBE coatings without and with the addition of SiO_2_ fillers, preliminary AC-DC-AC experiment was conducted by employing different DC excitation voltages upon the blank WBE coatings. As such, the Bode spectra obtained under ±2 V, ±3 V, and ±4 V cyclic DC polarization are shown in Figure 7. The terminated impedance values obtained at 10^−1^ Hz were used to evaluate the variation of the anti-corrosion performance of different systems. As shown in Figure 7a, an expired impedance of approximately 10^6^ Ω·cm^2^ is obtained after two cycles of ±2 V polarization, after which this impedance resistance value reduces to about one decade lower after the 4th cycle, and it remains with relatively limited changes until the end (10 cycles) of testing. The difference in impedance obtained between the 2nd and the following polarization cycles could be caused by the diffusion of corrosive species, which would move into the substrate through the inherent tiny pores, and the stable EIS test results could demonstrate that the coating without fillers is not damaged under this polarization condition. Increasing the cyclic excitation voltage to ±3 V, the impedance value drops to 10^5^ Ω·cm^2^ after two cycles polarization as shown in Figure 7b. In spite of this, the DC voltage of ±3 V is still not enough to quickly evaluate the corrosion resistance of the coating. A clear but not aggressive degradation of the coating without fillers is achieved by applying the excitation voltage of ±4 V as shown in Figure 7c. The terminated impedance values are 2.2 × 10^5^, 1.7 × 10^4^, 2.6 × 10^4^, and 4.7 × 10^4^ Ω·cm^2^ after 2nd, 4th, 6th, and 8th polarization, respectively, the results are also coupled with the gradual shift of the phase towards lower degrees. However, further increasing the polarization repetition to 10 cycles, the impedance increases to 8.3 × 10^4^ Ω·cm^2^ and a new time constant appears at the middle frequency region. A significant change in the Bode spectrum can be attributed to the corrosion of the metallic substrate, whose corrosion products conceal the defects caused by the polarization in the resin coatings [59]. With the consideration of the difference in EIS results obtained under different DC polarization values, the anti-corrosion performance of WBE resin coatings and composite coatings with different additions of SiO_2_ has been evaluated under ±4 V.

**Figure 8:**
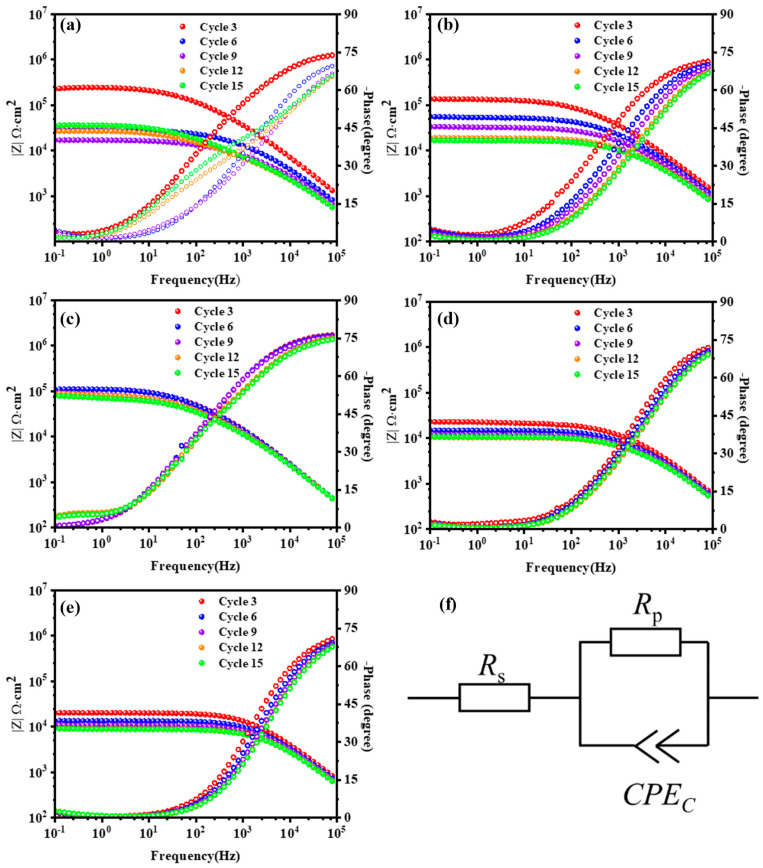
Bode plots of (**a**) EP-0; (**b**) EP-1; (**c**) EP-3; (**d**) EP-5; (**e**) EP-10 coatings during AC-DC-AC test. (**f**) the corresponding equivalent circuits.

Figure 8 depicts the Bode plots for the WBE resin coating and composite coatings containing different amounts of modified SiO_2_. For better comparison, the Bode impedance and phase plots of cycle 3, cycle 6, cycle 9, cycle 12, and cycle 15 DC bipolarization are illustrated. EP-0 obtained the highest impedance value of 2.2 × 10^5^ Ω·cm^2^ in the low frequency and also the highest phase angle −4.1° after the 3rd polarization of the AC-DC-AC test. But the impedance value dropped to the lowest value after the 9th polarization of the AC-DC-AC test, which is 1.6 × 10^5^ Ω·cm^2^. After the 12th and 15th polarizations, corrosion products filled in the damaged area of the coating, resulting in a slight increase in low frequency impedance. The phase angle displays a new time constant at the middle frequency region, which is due to interfacial corrosion caused by the corrosive medium penetrating the metallic substrate [59]. In addition to revealing significant fluctuations in EP-1 impedance at low frequencies, the AC-DC-AC tests also reveal significant phase angle fluctuations. Perhaps this is due to the fact that the addition of a small quantity of SiO_2_ cannot fully plug the pores that were produced during the coating cures. EP-3 maintained the best corrosion resistance, as shown in Figure 8c, and the impedance value in low frequency and the phase angle in high frequency were nearly constant after the 9th AC-DC-AC cycles, which are 9.4 × 10^5^ Ω·cm^2^ and −0.9°, respectively. Consequently, it is possible to demonstrate that the modified SiO_2_ in an appropriate amount could improve the corrosion resistance of the coating. Additionally, due to the high amounts of amino groups in KH550, cross-linking is enhanced during coating curing, which results in a higher coating density. It lacks a significant change in phase angle at high frequency, but the impedance value in low frequency of the samples decreased significantly within the same number of cycles when the SiO_2_ content increased to 5 wt.% and 10 wt.%. In comparison with other samples, EP-10 has the lowest impedance value and small flow during all AC-DC-AC tests. The impedance value of EP-10 in low frequency is 9.1 × 10^4^ Ω·cm^2^ after the 15th polarization, indicating that the sample maintains the lowest corrosion resistance. This may be due to an excessive amount of SiO_2_ causing the agglomeration of fillers, which leads to a reduction in the uniformity of the coating and an appearance of larger defects during curing.

**Figure 9:**
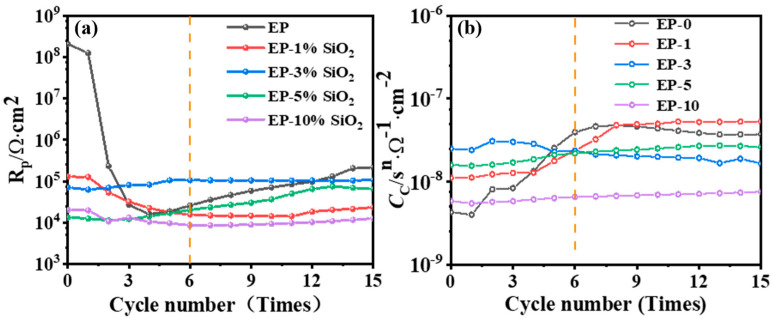
Changes in the characteristic parameters of the coating systems during the AC-DC-AC test. (**a**) the pore resistance; (**b**) The effective coating capacitor.

Based on the AC-DC-AC testing, Figure 9 shows the evolution of the coating’s characteristic parameters over the cycle times. In Figure 8f, an equivalent circuit model is presented, which can be used to interpret the experimental EIS data for pure and composite coatings on metallic substrates. *R*_s_ refers to the resistance of the electrolyte, which is the resistance between the reference electrode and working electrode. An electrolyte’s porous resistance (*R*_p_) is determined by the diameter of pores, the porosity, and the characteristics of capillary channels by which the ions penetrate an interface. In Figure 9a, the *R*_p_ changes of different samples are relatively different during the first six cycles. Relative to other samples, the *R*_p_ values of EP-0 and EP-1 are relatively high, but there is a large drop in the first four AC-DC cycles, suggesting that an aggressive species might have penetrated into the metal substrate surface of EP-0 and EP-1 due to there being more micropores on their surfaces [18]. The *R*_p_ values of EP-5 and EP-10 are low and show a gradual upward trend in the first six cycles. It was found that the *R*_p_ of EP-3 was changed by a small amount in comparison with other composite coatings during the 1st to 6th cycles. During the 6th to 15th cycles, the *R*_p_ values of EP-0, EP-1, EP-5, and EP-10 all increased slightly. The corrosion resistance of the coating is increased because the corrosion products produced during anodic polarization temporarily resist defects created during coating degradation [60]. However, during the 6th to 15th cycles, the *R*_p_ of EP-3 remains relatively stable, indicating that it has the best corrosion resistance. A greater number of electrolytes will be absorbed by the coating as moisture permeates into it, thus increasing the coating capacitance *C*_C_ [61]. In the electrical equivalent of Figure 8f, *C*_CPE, T_ and *C*_CPE, P_ are parallel to *R*_p_, making it possible to calculate its effective coating capacitance by using Equation (1):

(1) $$C_{C}=\frac{{\left(C_{\mathrm{CPE},T}R_{P}\right)}^{1/C_{\mathrm{CPE},P}}}{R_{P}}$$

where *R*_p_ is the resistance of the resistance parallel to the *C*_CPE,T_ and *C*_CPE,P._ All capacitance values presented were determined according to Equation (1) [62].

The changes of *C*_C_ of different samples in the AC-DC-AC test are shown in Figure 9b. The *C*_C_ of EP-0 gradually increases, indicating that the capacitive effect of the pure coating is increased. The *C*_C_ of EP-3 increased slightly during the 1st to 6th cycles but remained relatively stable overall, which indicates that no further reaction occurred at the interface between coating and metallic substrate after the coating reached water saturation. From the 6th to the 15th cycles, there is no significant change in the corrosion coefficient of all samples, indicating that the coating has reached saturation in terms of water uptake [63].

### 3.3. Corrosion Evaluation of Composite Coating

**Figure 10:**
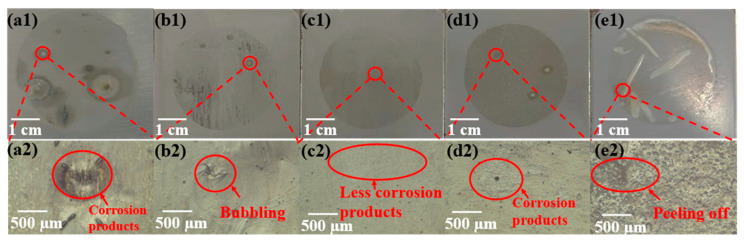
The surface morphologies of pure coating and composite coating with different content of SiO_2_ after AC-DC-AC test. (**a1**,**a2**) EP-0; (**b1**,**b2**) EP-1; (**c1**,**c2**) EP-3; (**d1**,**d2**) EP-5; (**e1**,**e2**) EP-10.

The surface morphology of the coating should be assessed upon completion of the AC-DC-AC test in order to determine the most appropriate amount of SiO_2_ to add to the coating. Based on the AC-DC-AC test results, it can be seen that pure coatings and composite coatings with different SiO_2_ content exhibit different surface morphologies as shown in Figure 10. According to Figure 10(a1,b1,d1), there are some bulges on the surface of the EP-0, EP-1, EP-5 coatings. Further observation of these areas of bubble formation through an optical microscope shows that there are corrosion products precipitated around these areas as shown in Figure 10(a2,b2,d2). Due to cathodic polarization, oxygen reduction and alkalinization are generated, which stretches out the coating surface, reduces the coating density, and facilitates the corrosive medium permeating around this large bulge, which in turn makes corrosion products more likely to accumulate around these areas during anodic polarization. Therefore, corrosion may first occur in these areas. As can be seen in Figure 10(e1), the coating is almost completely stripped from the metallic substrate, and there is a great deal of rusting products around the exfoliated coating as shown in Figure 10(e2). Through the cracks and voids of the composite coating, the corrosion medium had penetrated the metallic substrate. Cathodic polarization also produced oxygen reduction and alkalinization, which would exacerbate the degradation and spalling process of the coating. As illustrated in Figure 10(c1), corrosion damage on the coating surface is relatively slight, and there is almost no trace of corrosion damage as shown in Figure 10(c2). The EP-3 coating has the best barrier properties to aggressive media. The observed results of the coating surface morphology are consistent with the AC-DC-AC test results as shown in Figure 8a–e.

**Figure 11:**
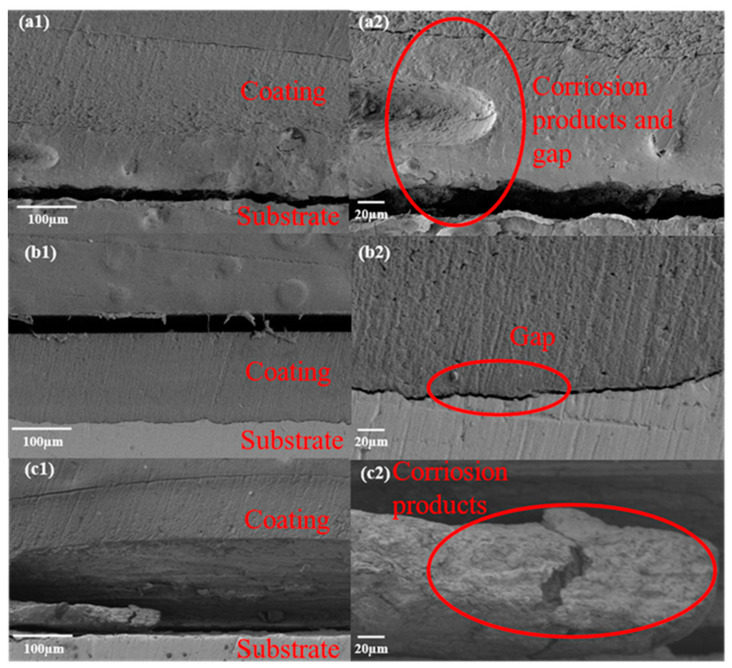
The cross-section morphology after AC-DC-AC test (**a1**,**a2**) EP-0; (**b1**,**b2**) EP-3; (**c1**,**c2**) EP-10.

According to the results of the AC-DC-AC test, the corrosion damage of the coating can be determined by detecting the blistering and peeling that takes place at the interface, which would be broken between the coating and the metallic substrate due to metallic substrate corrosion. Figure 11 shows the cross-sectional morphology of EP-0, EP-3, and EP-10. According to Figure 11(a1), the coating has been stripped off from the metallic substrate and some corrosion products have been stripped in the delamination. In Figure 11(a2), there is an obvious delamination between the corrosion product and the substrate, leading to a significant decrease in the adhesion of the coating. Figure 11(b1) indicates that there is no obvious corrosion product between the coating and the metallic substrate. Furthermore, there is a little delamination between the coating and the metallic substrate as shown in Figure 11(b2). In Figure 11(c1), there is an obvious bulge in the composite coating, and it is severely separated from the metallic substrate. It can be shown than there are many loose corrosions products deposited on the top of the metallic substrate as shown in Figure 11(c2), indicating that the interface was subject to serious corrosion. In comparison with other samples, from the cross-section morphology between the EP-3 coating and the metallic substrate, there was a slight peeling of the coating away from the metallic substrate but no obvious damage to the coating after the AC-DC-AC test, indicating a low degree of corrosion on the metallic substrate. By adding a sufficient and appropriate amount of SiO_2_ to the coating, the pores generated during the curing process of the coating can be effectively blocked, thereby increasing the difficulty for water molecules or other corrosive media to penetrate the coating and enter the metallic substrate.

**Figure 12:**
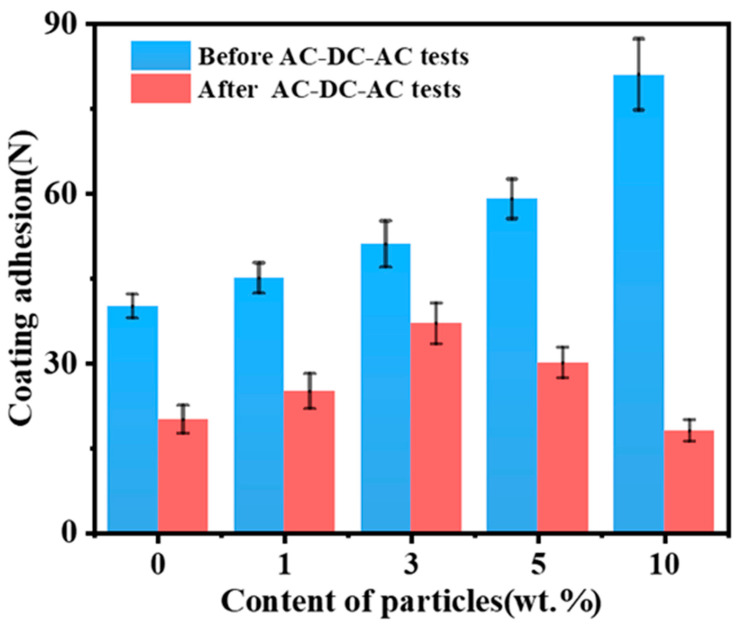
The adhesion of the interface between composite coating and metallic substrate before and after AC-DC-AC test.

The adhesion test results of composite coatings containing different amounts of SiO_2_ are shown in Figure 12. It is evident that adhesion strength increased with increased SiO_2_ content as shown in Figure 12. There is an average adhesion of 81 N for the EP-10 coating. This is due to the fact that SiO_2_ has a certain reinforcing effect as a hard phase, and it disperses widely on the coating’s surfaces. As a result of the interaction between particles and the active groups of the epoxy resin or curing agent, the coating is more tightly bonded with the metallic substrate, improving the coating’s adhesion. Compared with other samples, the adhesion between the EP-3 coating and the metallic substrate decreased the least after the AC-DC-AC test, which was only 37 N. However, the adhesion between the EP-10 coating and the metallic substrate decreased to 18 N. In this case, excessive SiO_2_ was added to the epoxy resin, resulting in an agglomeration at the interface that reduces the uniformity of the coating and causes larger defects. This, in turn, will result in more corrosion media entering the metallic substrate, resulting in more oxygen reduction and alkalinization being produced during the AC-DC-AC test. Cathodic polarization results in oxygen reduction and alkalinization, which accelerates coating spalling, resulting in a reduction of adhesion between the coating and the metallic substrate [62].

**Figure 13:**
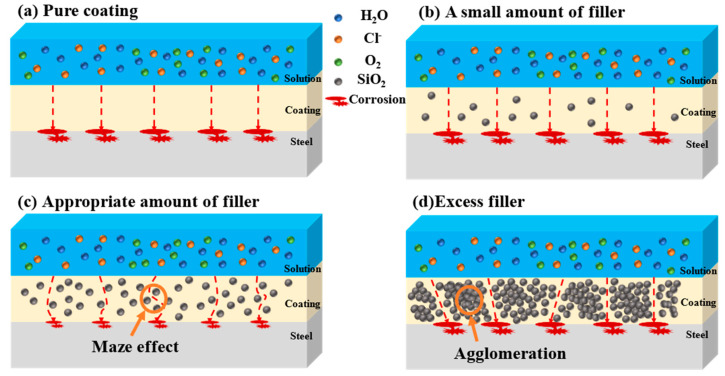
Schematic of the modified SiO_2_ dispersion on the barrier properties of coating (**a**) pure coating (**b**) small amount of SiO_2_ (**c**) appropriate amount of SiO_2_ and (**d**) excess SiO_2_.

The corrosion protection mechanism of composite coatings is shown in Figure 13. According to Figure 13a, a single water-borne epoxy coating is easily porous during curing, because of the large surface tension of water. This does not provide adequate barrier properties, which results in corrosion of the metallic substrate through the easy entry of corrosive media through micropores. Figure 13b shows that a small amount of SiO_2_ is not capable of effectively filling the pores in epoxy resin, which does not improve its anticorrosion performance. Figure 13c illustrates that when the coating is uniformly dispersed with an appropriate amount of SiO_2_, pores can be effectively plugged and corrosive media are less likely to penetrate. Consequently, the coating performs better as a barrier and is more corrosion resistant. In Figure 13d, the agglomeration of SiO_2_ will lead to the decrease of the specific surface area of fillers, resulting in greater defects in the interface between fillers and resin during volume shrinkage during resin curing, and the agglomerated fillers will also reduce the uniformity of the coating. Therefore, excessive water absorption of the composite coating, increased internal stress, and bubbling occur. The composite coating may lose its protective effect in the application process. The coating’s protective properties can, therefore, be increased by adding a suitable amount of SiO_2_ to the resin. It is possible to block micropores during curing by adding sufficient quantities of SiO_2_ to the coating. SiO_2_ has the characteristics of a large specific surface area, high specific surface energy, and strong activity, which enable it to facilitate the cross-linking reaction between epoxy resins and improve the coating’s compactness and mechanical properties [64,65,66]. It is as a result of this improvement in density that electrolyte permeation is slowed, thus increasing the coating’s protection and allowing it to last for a long period of time.

## 4. Conclusions

The AC-DC-AC test was performed in this study to accelerate corrosion damage in composite coatings containing micro-SiO_2_. According to the result, the following conclusions can be drawn:

(1) Spherical SiO_2_ particles with a particle size of 800 ± 50 nm were synthesized by a solution-gel method.
(2) By modifying SiO_2_ with the silane coupling agent KH550, appropriate amounts of SiO_2_ can be dispersed in water-borne epoxy resins more effectively.
(3) The corrosion resistance increases first with an increase in SiO_2_ content in the composite coating, and then decreases as SiO_2_ fills the pores formed in the WBE resin coating during curing. It is observed that the composite coating has the best corrosion resistance when the addition of SiO_2_ amounts to 3 wt.%; it has the lowest impedance value of 9.4 × 10^5^ Ω·cm^2^ after the AC-DC-AC test; and its adhesion decreases the least before and after the AC-DC-AC test.

## Figures and Tables

**Table 1:** Curing agent-related information.

BH-532	
Solid content (%)	45 ± 2
Amine hydrogen equivalent	220–280
VOC content (%)	<2
pH	9–10

**Table 2:** Epoxy resin-related information.

BH-653	
Solid content (%)	60 ± 2
Epoxy equivalent	180–230
Viscosity (mpa·s/25 °C)	<2
pH	2–7

**Table 3:** *T*_−50%_, *T*^d^_peak_, and remaining content of composite coatings with different contents of SiO_2_.

Sample	*T*_−50%_ (°C)	*T*^d^_peak_ (°C)	Remain Content (%)
EP-0	356.1	351.6	12.5
EP-1	357.6	352.1	14.6
EP-3	362.3	356.8	15.6
EP-5	369.4	357.4	17.2
EP-10	376.3	361.8	20.7

## Data Availability

The data presented in this study are available on request from the corresponding author.

## Supplementary Materials

The following supporting information can be downloaded at: https://www.mdpi.com/article/10.3390/polym15153273/s1, Figure S1: Dispersion of SiO_2_ with different contents in water. Figure S2: cross-section morphology of the composite coating (a) EP-0; (b) EP-1; (c) EP-3; (d) EP-5; (e) EP-10. Figure S3: Bode plots of (a) EP-0; (b) EP-1; (c) EP-3; (d)EP-5; (e) EP-10 coatings natural immersed 30 days in 3.5 wt.% NaCl solution.

## References

[1] Song D., Wan H., Tu X., Li W. (2020). A better understanding of failure process of waterborne coating/metal interface evaluated by electrochemical impedance spectroscopy. Prog. Org. Coat..

[2] Hamadi L., Mansouri S., Oulmi K., Kareche A. (2018). The use of amino acids as corrosion inhibitors for metals: A review. Egypt. J. Pet..

[3] Jin B., Xiong D.-B., Tan Z., Fan G., Guo Q., Su Y., Li Z., Zhang D. (2019). Enhanced corrosion resistance in metal matrix composites assembled from graphene encapsulated copper nanoflakes. Carbon.

[4] Li X., Zhang D., Liu Z., Li Z., Du C., Dong C.J.N. (2015). Materials science: Share corrosion data. Nature.

[5] Ambrosi A., Pumera M. (2015). The Structural Stability of Graphene Anticorrosion Coating Materials is Compromised at Low Potentials. Chemistry.

[6] Faccini M., Bautista L., Soldi L., Escobar A.M., Altavilla M., Calvet M., Domènech A., Domínguez E. (2021). Environmentally Friendly Anticorrosive Polymeric Coatings. Appl. Sci..

[7] Koch G.H., Brongers M.P., Thompson N.G., Virmani Y.P., Payer J.H. (2002). Corrosion Cost and Preventive Strategies in the United States; United States.

[8] Rodriguez A.A., Miller C.M., Monty C.N. (2021). Field Testing and Cost–Benefit Evaluation of Corrosion-Protective Coatings on Winter Maintenance Equipment in the State of Ohio. J. Cold Reg. Eng..

[9] Rammelt U., Reinhard G. (1992). Application of electrochemical impedance spectroscopy (EIS) for characterizing the corrosion-protective performance of organic coatings on metals. Prog. Org. Coat..

[10] Kalita D.J., Tarnavchyk I., Chisholm B.J., Webster D.C. (2021). Novel bio-based epoxy resins from eugenol as an alternative to BPA epoxy and high throughput screening of the cured coatings. Polymer.

[11] Zhang C., Huang K.-C., Wang H., Zhou Q. (2020). Anti-corrosion non-isocyanate polyurethane polysiloxane organic/inorganic hybrid coatings. Prog. Org. Coat..

[12] Gujjar S.V., Nadar N., Choudhary K., Hunashyal A.M., Shahapurkar K., Mujtaba M.A., Asadullah M., Soudagar M.E.M., Khan T.M.Y., Ismail K.A. (2022). Investigation of Various Coating Resins for Optimal Anticorrosion and Mechanical Properties of Mild Steel Surface in NaCl Solution. Adv. Mater. Sci. Eng..

[13] Hakeim O.A., Abdelghaffar F., Haroun A.A. (2020). UV-curable hyperbranched polyester acrylate encapsulation of phthalocyanine pigments for high performance synthetic fabrics printing. Dye. Pigment..

[14] Olajire A.A. (2018). Recent advances on organic coating system technologies for corrosion protection of offshore metallic structures. J. Mol. Liq..

[15] ESharmin, Imo L., Ashraf S.M., Ahmad S. (2004). Acrylic-melamine modified DGEBA-epoxy coatings and their anticorrosive behavior. Prog. Org. Coat..

[16] Grigoriev D., Shchukina E., Tleuova A., Aidarova S., Shchukin D. (2016). Core/shell emulsion micro- and nanocontainers for self-protecting water based coatings. Surf. Coat. Technol..

[17] Chi J., Zhang G., Xie Q., Ma C., Zhang G. (2020). High performance epoxy coating with cross-linkable solvent via Diels-Alder reaction for anti-corrosion of concrete. Prog. Org. Coat..

[18] Wang J., Du P., Zhao H., Pu J., Yu C. (2019). Novel nitrogen doped carbon dots enhancing the anticorrosive performance of waterborne epoxy coatings. Nanoscale Adv..

[19] Wang G., Zhou Z., Hu Q., Shi X., Zhang X., Zhang K., Wu L. (2023). Preparation of eco-friendly natural rosin-based SiO_2_–NH_2_@GO hybrid sealant and study on corrosion resistance of Fe-based amorphous coating for steel substrate. Carbon.

[20] Yan H., Cai M., Li W., Fan X., Zhu M. (2020). Amino-functionalized Ti3C2T with anti-corrosive/wear function for waterborne epoxy coating. J. Mater. Sci. Technol..

[21] Shibata M., Ishigami N., Shibita A. (2017). Synthesis of sugar alcohol-derived water-soluble polyamines by the thiol-ene reaction and their utilization as hardeners of water-soluble bio-based epoxy resins. React. Funct. Polym..

[22] Dong C.F., Sheng H., An Y.H., Li X.G., Xiao K., Cheng Y.F. (2010). Corrosion of 7A04 aluminum alloy under defected epoxy coating studied by localized electrochemical impedance spectroscopy. Prog. Org. Coat..

[23] Leal D.A., Kuznetsova A., Silva G.M., Tedim J., Wypych F., Marino C.E.B. (2022). Layered materials as nanocontainers for active corrosion protection: A brief review. Appl. Clay Sci..

[24] Lyon S.B., Bingham R., Mills D.J. (2017). Advances in corrosion protection by organic coatings: What we know and what we would like to know. Prog. Org. Coat..

[25] Tian W., Liu L., Meng F., Liu Y., Li Y., Wang F. (2014). The failure behaviour of an epoxy glass flake coating/steel system under marine alternating hydrostatic pressure. Corros. Sci..

[26] Xie Z.H., Li D., Skeete Z., Sharma A., Zhong C.J. (2017). Nanocontainer-Enhanced Self-Healing for Corrosion-Resistant Ni Coating on Mg Alloy. ACS Appl. Mater. Interfaces.

[27] Liu L., Zhao M., Pei X., Liu S., Luo S., Yan M., Shao R., Sun Y., Xu W., Xu Z. (2023). Improving corrosion resistance of epoxy coating by optimizing the stress distribution and dispersion of SiO2 filler. Prog. Org. Coat..

[28] Daradmare S., Raj S., Bhattacharyya A.R., Parida S. (2018). Factors affecting barrier performance of composite anti-corrosion coatings prepared by using electrochemically exfoliated few-layer graphene as filler. Compos. Part B Eng..

[29] Tong Y., Bohm S., Song M. (2017). The capability of graphene on improving the electrical conductivity and anti-corrosion properties of Polyurethane coatings. Appl. Surf. Sci..

[30] Aung M.M., Li W.J., Lim H.N. (2020). Improvement of anticorrosion coating properties in bio-based polymer epoxy acrylate incorporated with nano zinc oxide particles. Ind. Eng. Chem. Res..

[31] Wang F., Feng L., Lu M. (2019). Mechanical properties of multi-walled carbon nanotube/waterborne polyurethane conductive coatings prepared by electrostatic spraying. Polymers.

[32] Wu Z., Cao S., Sun Q., Zhong F., Zhang M., Duan H. (2021). Technology, Mechanical, thermal and gas sensing properties of flexible multi-walled carbon nanotubes/waterborne polyurethane composite film. Compos. Sci. Technol..

[33] Alrashed M.M., Soucek M.D., Jana S.C. (2019). Role of graphene oxide and functionalized graphene oxide in protective hybrid coatings. Prog. Org. Coat..

[34] Wang Y., Wang H., Li Z., Yang D., Qiu X., Liu Y., Yan M., Li Q. (2021). Fabrication of litchi-like lignin/zinc oxide composites with enhanced antibacterial activity and their application in polyurethane films. J. Colloid Interface Sci..

[35] Nguyen T.M., Bui T.M.A., Nguyen T.V. (2020). Acid and alkali resistance of Acrylic polyurethane/R-SiO_2_ nanocomposite coating. Vietnam. J. Chem..

[36] Borisova D., Möhwald H., Shchukin G.M. (2011). Mesoporous Silica Nanoparticles for Active Corrosion Protection. ACS Nano.

[37] Knudsen O.Ø., Forsgren A. (2017). Corrosion Control through Organic Coatings.

[38] Jena G., George R.P., Philip J. (2021). Fabrication of a robust graphene oxide-nano SiO_2_-polydimethylsiloxane composite coating on carbon steel for marine applications. Prog. Org. Coat..

[39] Wang J., Zhang L., Li C. (2022). Superhydrophobic and mechanically robust polysiloxane composite coatings containing modified silica nanoparticles and PS-grafted halloysite nanotubes. Chin. J. Chem. Eng..

[40] Lu Z., Xu L., He Y., Zhou J. (2019). One-step facile route to fabricate functionalized nano-silica and silicone sealant based transparent superhydrophobic coating. Thin Solid Films.

[41] Li W., Tian H., Hou B. (2012). Corrosion performance of epoxy coatings modified by nanoparticulate SiO_2_. Mater. Corros..

[42] Palraj S., Selvaraj M., Maruthan K., Rajagopal G. (2015). Corrosion and wear resistance behavior of nano-silica epoxy composite coatings. Prog. Org. Coat..

[43] Ke Q., Fu W., Jin H., Zhang L., Tang T., Zhang J. (2011). Fabrication of mechanically robust superhydrophobic surfaces based on silica micro-nanoparticles and polydimethylsiloxane. Surf. Coat. Technol..

[44] Anitha C., Azim S.S., Mayavan S. (2018). Compounds, Influence of particle size in fluorine free corrosion resistance superhydrophobic coating-optimization and stabilization of interface by multiscale roughness. J. Alloy. Compd..

[45] Cao M., Jin M., Qiu J., Zhao X., Liu Y., Zhang X., Cai Q., Zhu W. (2014). The synthesis of a core–shell hybrid composite micro-sphere with controllable homogenous or heterogeneous multi-shell structure by multiple-growth via a combination method. Colloids Surf. A Physicochem. Eng. Asp..

[46] Khouchaf L., Boulahya K., Das P.P., Nicolopoulos S., Kis V.K., Lábár J.L. (2020). Study of the microstructure of amorphous silica nanostructures using high-resolution electron microscopy, electron energy loss spectroscopy, X-ray powder diffraction, and electron pair distribution function. Materials.

[47] Tomiyama S., Takahashi R., Sato S., Sodesawa T., Yoshida S. (2003). Preparation of Ni/SiO_2_ catalyst with high thermal stability for CO_2_-reforming of CH_4_. Appl. Catal. A Gen..

[48] Stöber W., Fink A., Bohn E. (1968). Controlled growth of monodisperse silica spheres in the micron size range. J. Colloid Interface Sci..

[49] Lippincott E.R., Van Valkenburg A., Weir C.E., Bunting E. (1958). Infrared studies on polymorphs of silicon dioxide and germanium dioxide. J. Res. Natl. Bur. Stand..

[50] Šontevska V., Jovanovski G., Makreski P. (2007). Minerals from Macedonia. Part XIX. Vibrational spectroscopy as identificational tool for some sheet silicate minerals. J. Mol. Struct..

[51] Giordano L., Ricci D., Pacchioni G., Ugliengo P. (2005). Structure and vibrational spectra of crystalline SiO_2_ ultra-thin films on Mo (112). Surf. Sci..

[52] Huang J.Q., Liu K., Song X., Zheng G., Chen Q., Sun J., Jin H., Jiang L., Jiang Y., Zhang Y. (2022). Incorporation of Al_2_O_3_, GO, and Al_2_O_3_@GO nanoparticles into water-borne epoxy coatings: Abrasion and corrosion resistance. RSC Adv..

[53] Wei B., Chang Q., Bao C., Dai L., Zhang G., Wu F. (2013). Surface modification of filter medium particles with silane coupling agent KH550. Colloids Surfaces A Physicochem. Eng. Asp..

[54] Chen H., Lu H., Zhou Y., Zheng M., Ke C., Zeng D. (2012). Stability, Study on thermal properties of polyurethane nanocomposites based on organo-sepiolite. Polym. Degrad. Stab..

[55] Parida S.K., Dash S., Patel S., Mishra B.K. (2006). Adsorption of organic molecules on silica surface. Adv. Colloid. Interface Sci..

[56] Couchman P., Karasz F.J.M. (1978). A classical thermodynamic discussion of the effect of composition on glass-transition temperatures. Macromolecules.

[57] Moynihan C.T., Easteal A.J., Wilder J., Tucker J. (1974). Dependence of the glass transition temperature on heating and cooling rate. J. Phys. Chem..

[58] Hakkarainen M., Albertsson A.C., Karlsson S. (1996). Weight losses and molecular weight changes correlated with the evolution of hydroxyacids in simulated in vivo degradation of homo- and copolymers of PLA and PGA. Polym. Degrad. Stab..

[59] Yuan X., Yue Z.F., Chen X., Wen S.F., Li L., Feng T. (2015). EIS study of effective capacitance and water uptake behaviors of silicone-epoxy hybrid coatings on mild steel. Prog. Org. Coat..

[60] Molina J., Puig M., Gimeno M.J., Izquierdo R., Gracenea J.J., Suay J.J. (2016). Influence of zinc molybdenum phosphate pigment on coatings performance studied by electrochemical methods. Prog. Org. Coat..

[61] Meng F., Liu L., Tian W., Wu H., Li Y., Zhang T., Wang F. (2015). The influence of the chemically bonded interface between fillers and binder on the failure behaviour of an epoxy coating under marine alternating hydrostatic pressure. Corros. Sci..

[62] da Silva Lopes T., Lopes T., Martins D., Carneiro C., Machado J., Mendes A. (2020). Accelerated aging of anticorrosive coatings: Two-stage approach to the AC/DC/AC electrochemical method. Prog. Org. Coat..

[63] Zhang J., Hu J., Zhang J., Cao C. (2004). Studies of impedance models and water transport behaviors of polypropylene coated metals in NaCl solution. Prog. Org. Coat..

[64] Lin H., Wang Y. (2023). An organic phosphonic acid doped polyaniline/zirconia/epoxy composite coating for metal protection in the marine environment. Prog. Org. Coat..

[65] Chen J., Zhao W. (2021). Silk fibroin-Ti_3_C_2_T_X_ hybrid nanofiller enhance corrosion protection for waterborne epoxy coatings under deep sea environment. Chem. Eng. J..

[66] Sun J., Li W., Li N., Zhan Y., Tian L., Wang Y. (2022). Effect of surface modified nano-SiO_2_ particles on properties of TO@CA/SR self-healing anti-corrosion composite coating. Prog. Org. Coat..

