# Supplementary material for: Revealing the Impact of Micro-SiO2 Filer Content on the Anti-Corrosion Performance of Water-Borne Epoxy Resin
Source: Polymers (Basel). 2023 Aug 2;15(15):3273. doi: 10.3390/polym15153273 (PMC10422263; doi:10.3390/polym15153273)
Supplement: Supplementary file 1 [file polymers-15-03273-s001.zip › polymers-2513392-SI.pdf]

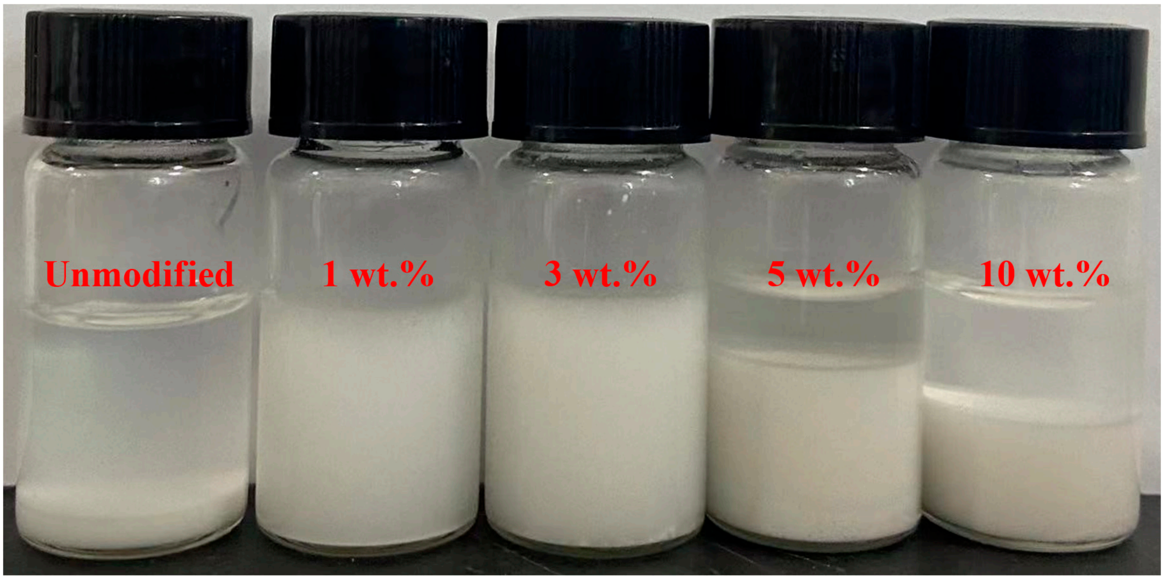

Fig. S1 Dispersion of SiO_2 with different contents in water.

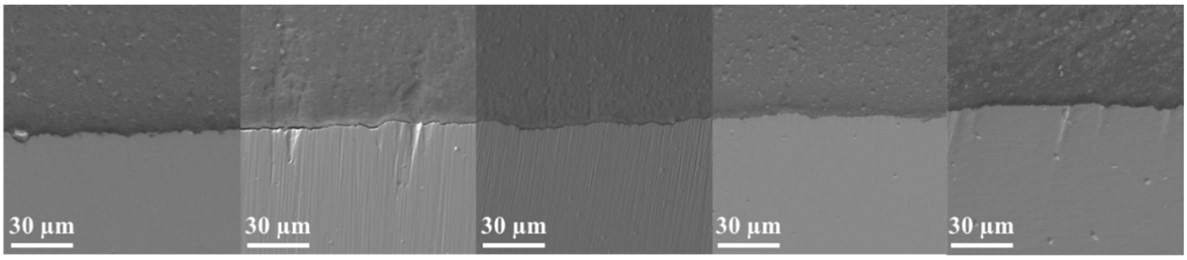

Fig. S2 cross-section morphology of the composite coating (a) EP-0; (b)EP-1; (c) EP-3; (d) EP-5; (e) EP-10.

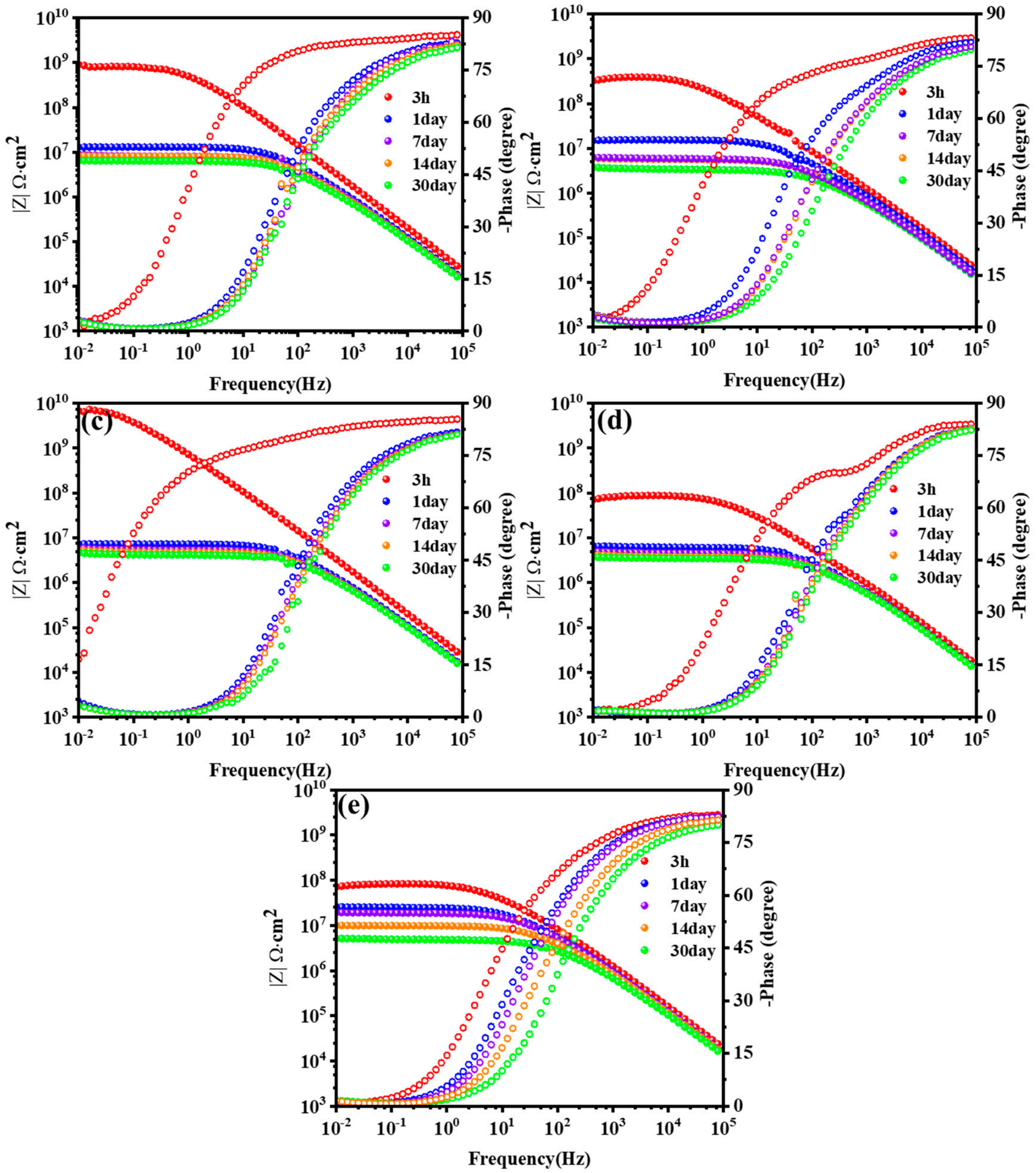

Fig. S3 Bode plots of (a)EP-0; (b)EP-1; (c)EP-3; (d)EP-5; (e)EP-10 coatings natural immersed 30 days in 3.5 wt.% NaCl solution.
